# Spatial distribution and losses by grain destroying insects in transgenic corn expressing the toxin Cry1Ab

**DOI:** 10.1371/journal.pone.0201201

**Published:** 2018-08-10

**Authors:** Gerson Adriano Silva, Izailda Barbosa Santos, Silvério Oliveira Campos, Tarcísio Visintin Silva Galdino, Elisângela Gomes Fidelis Morais, Júlio Claudio Martins, Lino Roberto Ferreira, Raul Narciso Carvalho Guedes, Marcelo Coutinho Picanço

**Affiliations:** 1 Departamento de Fitotecnia, Universidade Federal de Viçosa, Viçosa, Minas Gerais, Brazil; 2 Departamento de Entomologia, Universidade Federal de Viçosa, Viçosa, Minas Gerais, Brazil; Chinese Academy of Agricultural Sciences Institute of Plant Protection, CHINA

## Abstract

Insect pests are one of the factors that most impact plant yield. The magnitude of the losses and the spatiotemporal pest distribution in crops is a result of their interactions with the environment. Therefore, the understanding of the causes of production losses and the pest spatial patterns is important for the development of suitable sampling plans and pest management programs. Thus, this study aimed to quantify grain losses caused by insects and to determine the spatial distribution pattern of arthropod pest species in Bt and non-Bt corn. The prevailing insect pests in the corn ears were the earworm and fall armyworm caterpillars (*Helicoverpa* spp. and *Spodoptera frugiperda)*, the cornsilk fly (*Euxesta* spp.), the maize weevil (*Sitophilus zeamais*), and the square-necked grain beetle (*Cathartus quadricollis*). The non-Bt corn was more attacked by the caterpillars and the weevil, while Bt corn was more affected by the cornsilk fly *Euxest*a spp. Spatial dependence was significant for the damage caused by the caterpillars, the grain beetle and the maize weevil in both the Bt and non-Bt corn genotypes. The range of the damage caused by the insects was between 9.0–9.7 m for the caterpillars, 6.9–12.20 m for the cornsilk fly, 10.7–80.4 m for the square-necked grain beetle, and 51.9–170.7 m for the maize weevil. The pattern of the spatial distribution of pest damage in both corn genotypes (i.e., Bt and non-Bt corn) was similar with a prevalence of moderate to strong spatial dependence and aggregate damage distribution. The plants near to the sampling points exhibited injury and infestation levels similar to those of the sampled plants.

## Introduction

Insect pests can impact corn production by reducing the stand and plant production capacity, and corn quality by making corn ears unmarketable. Damage to marketable structures directly affects crop yield [1–3], and ear losses are the most significant impact on total corn crop losses. These losses can be caused by insect pests, physiological plant disorders, fungi and rodents [2,4,5]. In the field, the losses may occur from the beginning of the ear development of grains until the grain harvest. The most important insect pests during the early stages of the ear development are the fall armyworm *Spodoptera frugiperda*, the earworms *Helicoverpa* spp. (Lepidoptera: Noctuidae), the European corn borer *Ostrinia nubilalis* (Lepidoptera: Cambridae), and the cornsilk fly *Euxesta* spp. (Diptera: Otitidae) [6–8]. These pest species directly compromise corn yield due to silk consumption (which causes the abortion of ovules) and the grain consumption as well, and can also indirectly affect production while favoring fungal infestation and attacks by other insects on the damaged ears [9,10].

The control of armyworm, earworm and cornsilk fly in the corn ears is not effective. Nonetheless, many farmers use insecticides in such attempt and even at short time intervals (24–48 hours) [11–13], but the quick movement of neonates of these pest species to the apex of corn ears soon after the eggs hatch provide only partial insecticide coverage and short-term effectiveness with the application of these compounds [14]. As a result, the use of resistant crop varieties is considered an important control alternative and the development of genetically modified plants resistant to insects is in the forefront of this effort [15]. Genes of the bacteria *Bacillus thuringiensis* (Bt) expressing insecticidal proteins were introduced into corn plants, yielding transgenic corn (Bt corn) conferring resistance on certain caterpillars (Lepidoptera), beetles (Coleoptera), and flies (Diptera) regarded as pest species [16–18]. However, the landscape structure does have management consequences.

The success of pest control is influenced by the landscape structure and how organisms interact spatially and temporally with heterogeneous landscapes [19,20]. This recognition has been one of the new frontiers of ecology and has been increasingly explored from the perspective of pest management [21,22]. Annual crops represent temporary landscape fragments and influence the colonization, movement and distribution of pests. This reinforces the need for recognizing the matching patterns of spatial distribution of the associated pest species, which can be classified as aggregated, random or uniform. These patterns result from the interaction between the insect and the environment [23], and are influenced by the habitat quality [24]. Thus, when insects face habitats with food or shelter scarcity, difficulty mating, or unfavorable microclimate conditions, they migrate seeking better habitat conditions [24]. As a consequence, the knowledge about spatial distribution patterns of insect pests enables us to concentrate sampling efforts and management in areas with higher densities of these species [25].

The spatial distribution patterns of insects are often predicted using frequency distribution models. In these models, the data adjustment to the frequency distribution (e.g., negative binomial, Poisson or binomial positive) indicates that the spatial distribution patterns of insects are either aggregated, random or uniform [23]. However, such frequency distribution models are based on the average/variance ratio and do not consider the actual spatial distribution. Although this relationship ratio is affected by the spatial distribution, it does not represent the distribution of the individual in space [26]. One possibility for determining the factual spatial distribution pattern is through the use of geostatistical models [27].

Geostatistic verify whether the observed value of a variable for a given location is dependent on the values of neighboring sites. If there is spatial dependence, the variable displays spatial autocorrelation. For the modeling of spatial dependence in entomological studies with geostatistics, the semivariance function is used where semivariograms constitute adequate models to measure the pattern of spatial distribution of insects [21,25,28]. The recognition of such distribution allows the subsequent development of more suitable sampling plans and more efficient and precise management strategies. The sampling of armyworms, earworms and cornsilk flies usually relies on injury assessments in the field, while sampling of grain beetles is usually restricted to storage units after harvest. However, grain infestation usually takes place in the field, before harvest, particularly in warmer climates requiring the field assessment of the species spatial distribution patterns and dispersion before infestation in the storage units for their sound management. This would minimize the transportation of these insects and possible contamination of storage units. Therefore, the objective of this study was to determine the losses and spatial distribution of damage caused by insects that attack the ears of Bt (Cry1Ab) and non-Bt corn plants, such as caterpillars (*S*. *frugiperda* and *Helicoverpa* spp.), cornsilk flies and grain borer insects, on Bt (Cry1Ab) and non-Bt corn crops.

## Material and methods

### General field characterization

This study was conducted in the County of Cajuri, (20°47'27"S, 42°47'49"W; 678.84 m high), State of Minas Gerais, in Bt and non-Bt corn crops, during two agricultural seasons (2008/2009 and 2009/2010). The corn varieties used were DKB 390 YG (expressing the Bt protein Cry1Ab), which is to caterpillars, and its isoline DKB 390 (non-Bt). The spacing was 0.7 m between rows and 0.20 m between plants. The basic application of fertilizer was 500 kg/ha of NPK (8-24-12) and 500 kg/ha of the fertilizer 30-0-10 used in side-dressing splitted into two applications [1]. The cultivation techniques and phytosanitary control performed were those recommended for corn according to Galvão and Miranda [29].

### Sampling of insects and their damage

Insect sampling was performed at every 8m along the row and 10 m between the rows. At each sampling point, five ears were collected: one central, two in the neighboring plants in the same row and two in the neighboring plants on the side rows. In the first season, 307 points were established and 1535 ears were collected in Bt corn, while 280 points were established and 1400 ears were collected in non-Bt corn. In the second season, 405 and 368 points were established, and 2025 and 1840 ears were collected in Bt and non-Bt corn respectively. The collected ears were placed in plastic bags (five ears for each bag) and taken to the laboratory where the number of grains damaged by insects were determined; the ears were harvested with moisture content between 12 and 15% (w.b.) [30]. The damage caused by the earworm and armyworm caterpillars (*Helicoverpa* spp. and *S*. *frugiperda*) were pooled and regarded together as damage caused by caterpillars. The cornsilk fly attacks were recognized by the sole presence of the grain pericarp with the whole consumption of only the grain interior [31], in contrast with the damage by caterpillars, which included both grain interior and pericarp. Attacks by the grain beetles were recognized by the presence of larvae, adults and (associated) damaged corn grains. The insects causing damage to the grains were separated into morphospecies and stored in 70% ethanol solution for subsequent identification.

### Conventional statistical analysis of grain losses

The determination of losses was carried out for the insect species exhibiting frequency of occurrence above 10% in the samples from both corn genotypes and during the two agricultural seasons (S1 Table). The grain losses were estimated as a percentage of damaged grains in each ear, which was subjected to the non-parametric analysis of Kruskal-Wallis and the multiple comparisons were performed by Dunn's test *(P* < 0.05). Losses in Bt and non-Bt corn caused by each species or group of insects were compared by the Mann-Whitney U-test for two groups. A correlation analysis was also performed testing the association between the grain losses caused by caterpillars and those caused by the most frequent arthropod species (S1 Table). These statistical analyses were performed using the software SigmaPlot 12.5 (Systat, San Jose, CA, USA).

### Geostatistical analyses

The threshold of interference between sampling sites was determined using semivariogram models where the distance between sampling sites was the independent variable (x), and the corresponding semivariance used as dependent variable (y) was estimated using the formula:

$$\gamma (h)=\frac{1}{2N(h)}\sum {\left(ci-cj\right)}^{2},\;\text{where}:$$

*N(h)* is the number of pairs of sampling points separated from each other by a h distance, *ci* is the number of damaged grains from the first pair of sampling points and *cj* is the number of damaged grains from the second pair of sampling points, taken two by two.

A few important semivariogram parameters were also calculated, including: sill (C), nugget effect (C_o_) and range (A_o_) [32]. Subsequently, the anisotropy or isotropy of the sample data distribution was determined using semivariogram parameters [21]; when the semivariogram is identical for any direction of h, it is called isotropic, and when the semivariogram presents the parameters C, C_0_ and A_0_ or a differentiated model depending on the direction of h, it is called anisotropic. If isotropy occurs, a single semivariogram is adjusted, otherwise a different semivariogram must be adjusted for each direction.

The selection of the semivariogram model was performed by cross-validation using all sampling points. The parameters β_0_ (intercept) and β_1_ (slope) were obtained from the adjusted linear regression model of the observed values as a function of the estimated values and the better models are those with β_0_ and β_1_ closer to 0 and 1, respectively [33]. In addition, the residual sum of squares (RSS) and the coefficient of determination (R^2^) of the regression were also used for the semivariogram model selection. The lower the RSS value and the closer the R^2^ was to 1, the better the model [34]. The degree of spatial dependence (DSD) was estimated calculating the ratio between the nugget effect and the sill (DSD = C_0_/(C_0_+C)). The spatial dependence was considered strong when the estimated values were lower than or equal to 0.25, moderate when the values were between 0.25 and 0.75, and weak when the values were greater than 0.75.

After determining the spatial dependence between samples, the Kriging approach was used to construct the distribution maps of caterpillar damage (*S*. *frugiperda* + *Helicoverpa* spp.), cornsilk fly damage, and grain beetle damage [35]. The spatial analyses were performed using the GS+ program version 9.0 (Geostatistics for the Environmental Sciences, Gamma Design Software, LLC).

## Results

### Corn genotype losses

Species and groups present at frequencies higher than 10% were used to estimate the resulting grain the respective insect attacks and associated spatial distribution. These insects were: the earworm and armyworm caterpillars (*Helicoverpa* spp. e *Spodoptera frugiperda*), whose data was pooled together and analyses as damage due to “caterpillars”, the cornsilk fly *Euxesta* spp., the square-necked grain beetle *C*. *quadricollis*, and the maize weevil *S*. *zeamais* (Table 1).

**Table 1:** Frequency of damage caused by insects and mice to Bt (Cry1Ab) and non-Bt corn ears.

Pest	Frequency (%)			
	1st Year (2009/2010)		2nd Year (2011/2012)	
	Bt	Non-Bt	Bt	Non-Bt
*Cathartus quadricollis*	63.5	66.0	91.8	91.0
*Cryptolestes* sp.	0.4	1.6	8.4	6.8
*Euxesta* spp.	42.2	32.0	38.7	22.5
*S*. *zeamais*	10.5	15.2	61.7	65.7
*Tribolium* sp.	0.4	0.6	4.4	8.9
Caterpillars	77.9	82.5	85.7	94.3
Mice	4.3	7.1	2.0	1.4

**Fig 1:**
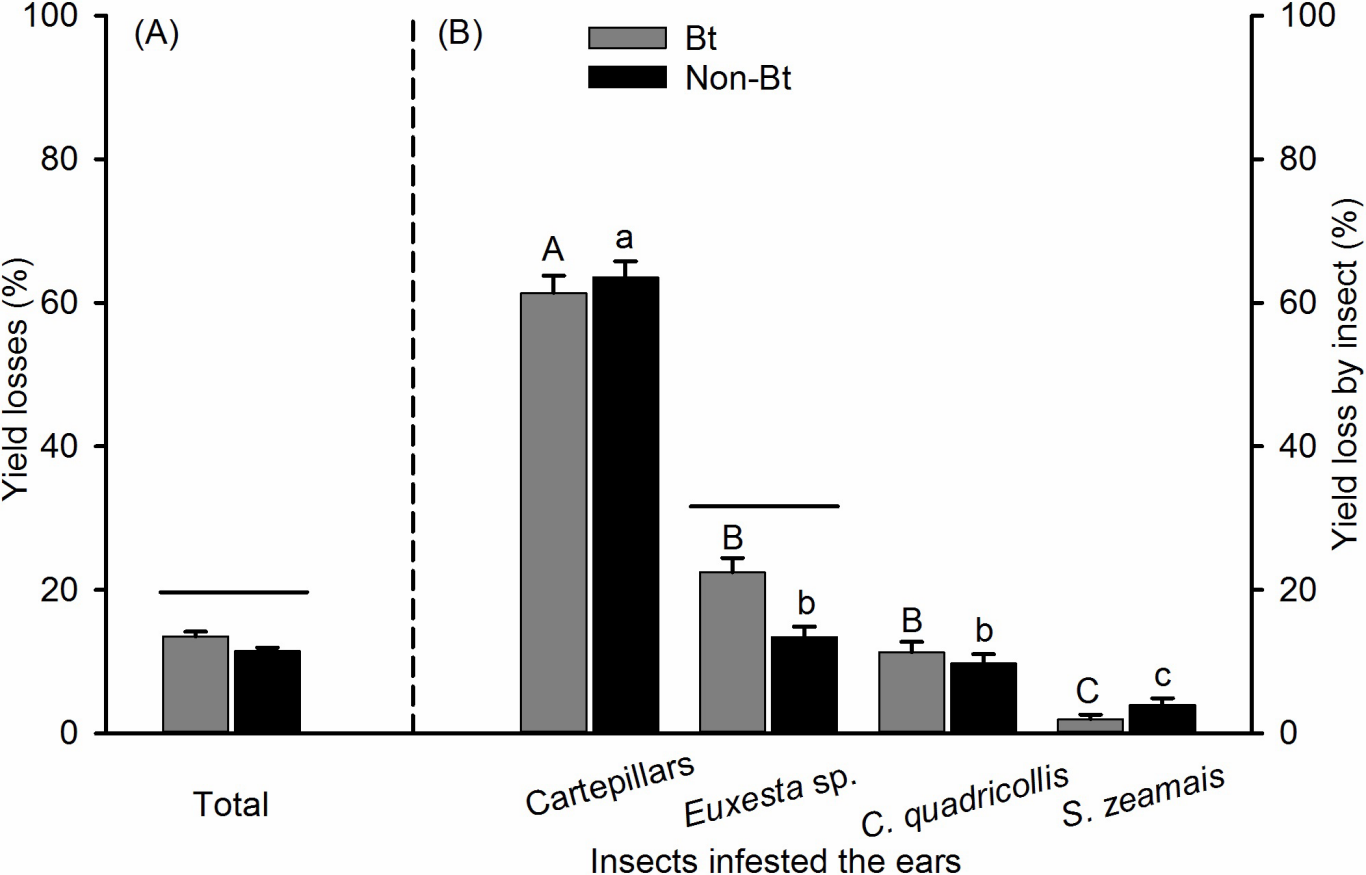
Mean losses (± SE) caused by insects in Bt and non-Bt corn in the first season of cultivation. (A) Total yield losses caused by insects, (B) yield losses caused by each insect within the total yield losses by insects. Means followed by the same letter are similar according to Dunn's test (*P* < 0.05); a capital letter represents grain losses of Bt corn; lowercase letters represent grain losses of non-Bt corn. A line represents a significant difference between Bt and non-Bt corn means by Mann-Whitney *U*-test for two groups (*P* < 0.05).

In the first agricultural season, the total losses caused by insects were higher in Bt (13.5%) than in non-Bt corn (11.4%) (Mann–Whitney *U* = 86672.5, *P* = 0.01) (Fig 1A). The damage caused by caterpillars, cornsilk fly, square-necked beetle and maize weevil *significantly differed in* Bt corn (Kruskal–Wallis *H*  =   338.7, df  =  3, *P* <  0.001), and also on non-Bt corn (Kruskal–Wallis *H*   =   409.5, df  =  3, *P* <  0.001). In both corn genotypes tested, the highest losses were caused by caterpillars (Bt = 61.4% and non-Bt = 63.5%), and the lowest by maize weevils (2.0 and 3.9%). The losses between corn genotypes were significantly different only for the cornsilk fly, which were higher on Bt (22.1%) than on non-Bt maize (13.4%) (Mann–Whitney *U* = 86889.0, *P* = 0.004) corn (Fig 1B).

**Fig 2:**
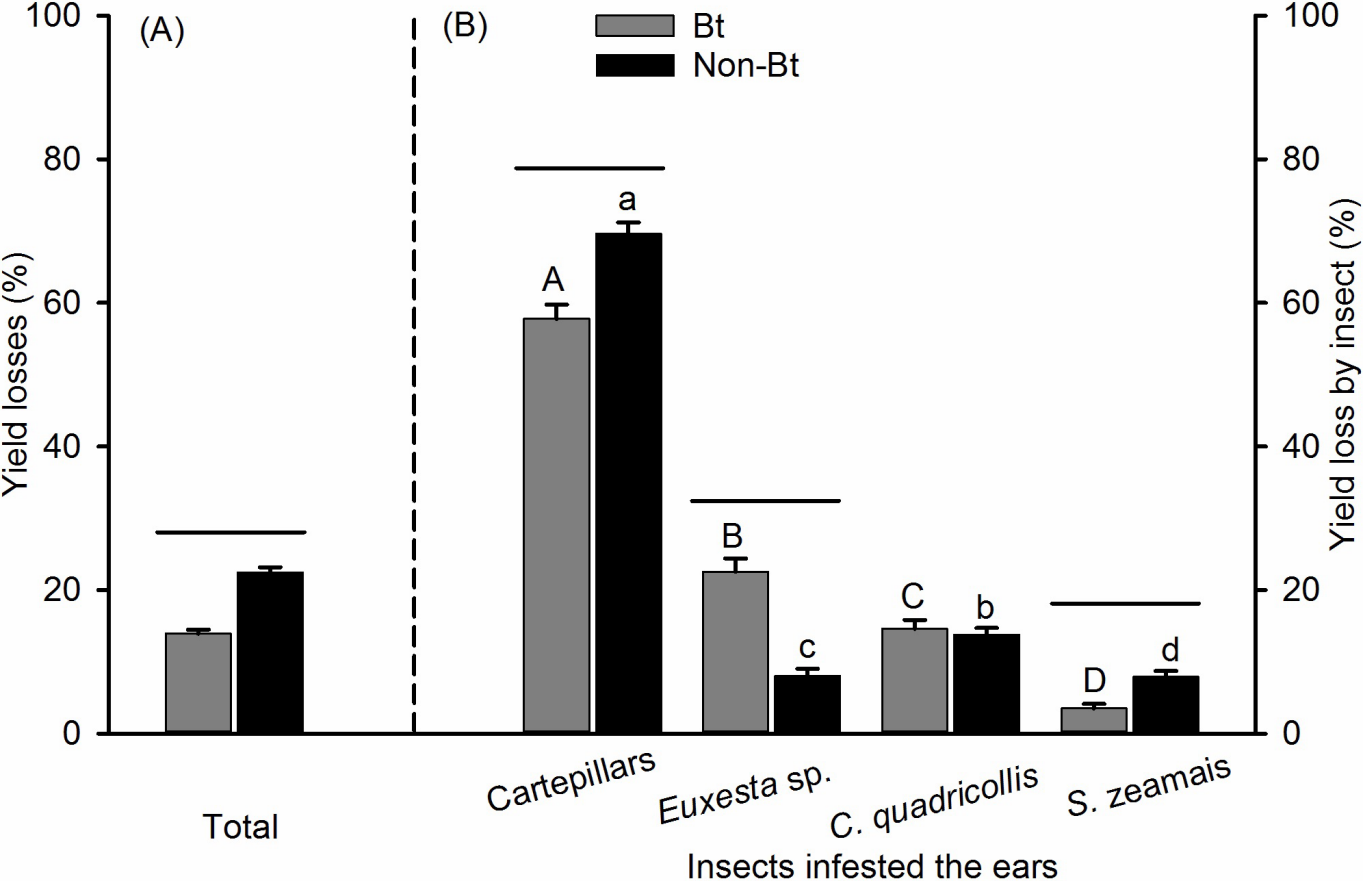
Mean losses (± SE) caused by insects in Bt and non-Bt corn in the second season of cultivation. (A) Total yield losses caused by insects, (B) yield losses caused by each insect within the total yield losses by insects. Means followed by the same letter are similar according to Dunn's test (*P* < 0.05); a capital letter represents grain losses of Bt corn; and a lowercase letter represents grain losses of non-Bt corn. A line represents a significant difference between Bt and non-Bt corn means according to Mann-Whitney U-test for the two groups (*P* < 0.05).

In the second agricultural season, the total Bt corn losses (13.9%) were lower than those in non-Bt corn (Mann–Whitney *U* = 117186.0, p < 0.001) (22.4%) (Fig 2A), as also recorded for the first season. The grain losses by insects species (or groups) also differed within each corn genotype with highest losses caused by caterpillars (Bt = 57.8%, non-Bt = 69.6%), regardless of corn genotype. Significant losses between corn genotypes were observed for all species, but the square-necked beetle, with higher losses by caterpillars and maize weevils on non-Bt corn and higher losses on Bt corn caused by the cornsilk fly (Mann–Whitney U ≥ 42125.0, *P* ≤ 0.002) (Fig 2B). The ears of corn attacked by caterpillars were also more attacked by the square-necked grain beetle and the maize weevil, while the cornsilk fly avoided corn avoided ears attacked by caterpillars (Table 2).

**Table 2:** Pearson’s correlation between grains damaged by caterpillar and by other insect pests in field corn, regardless of season and corn genotype.

Species	Grains damaged by caterpillar		
	Correlation coefficient (r)	t value	Significance (p)
*Cathartus quadricollis*	0.07	2.53	0.010*
*Cryptolestes* sp.	0.01	0.24	0.405
*Euxesta* sp.	-0.16	5.59	<0.001*
*Sitophilus*. *zeamais*	0.07	2.42	0.007*
*Tribolium* sp.	0.01	0.25	0.400
Mice	-0.02	0.77	0.220

* Significant to p < 0.05

### Spatial-dependence

**Fig 3:**
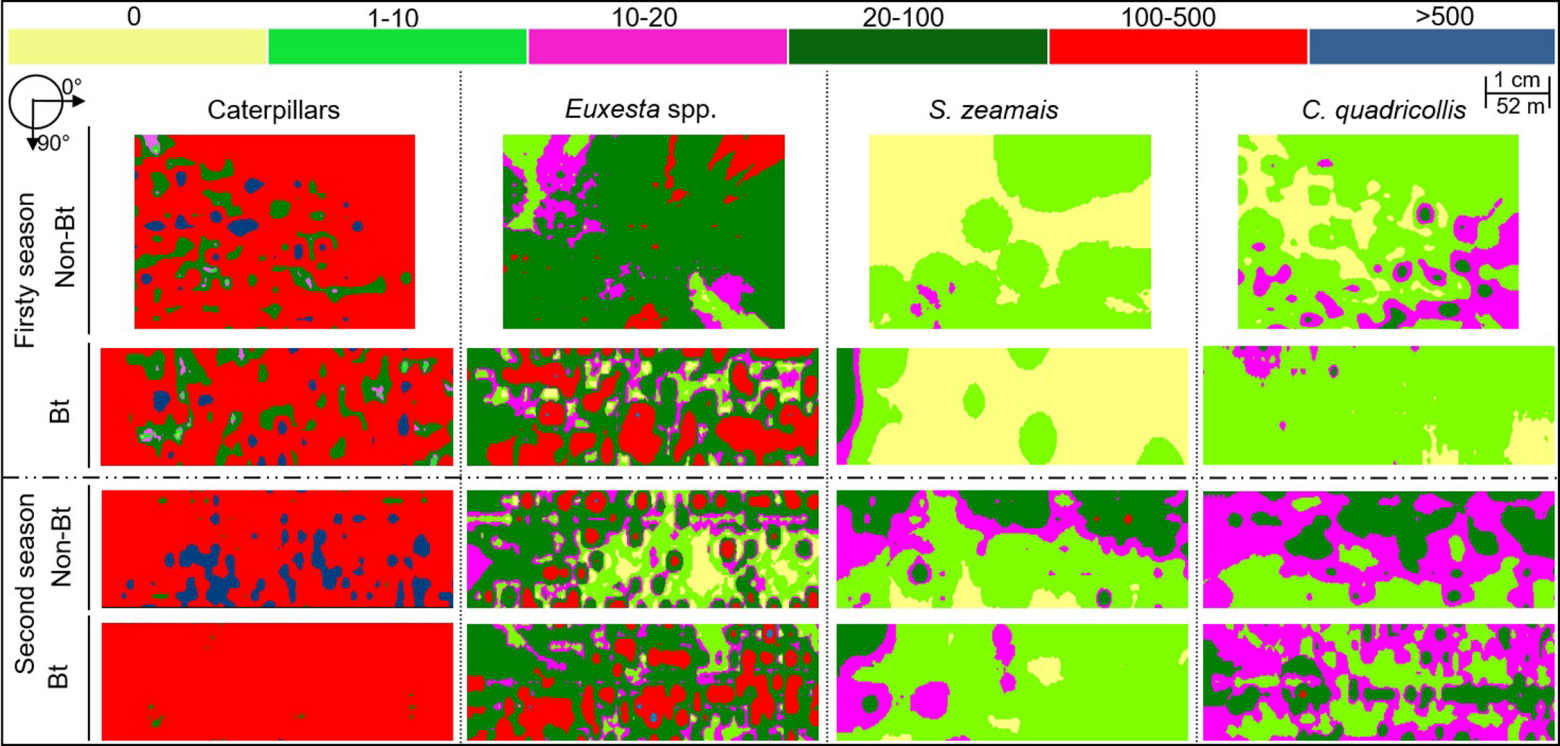
Spatial distribution of corn injury by caterpillars (*Helicoverpa* spp. + *Spodoptera frugiperda*), the cornsilk fly (*Euxesta* sp.), square-necked grain beetle (*C*. *quadricollis*), and the maize weevil (*S*. *zeamais*) in Bt (Cry1Ab) and non-Bt corn crops in the 2008/09 and 2009/10 seasons. Angle 0° = along the corn row and 90° = direction between corn rows. The maps are from infestations in Cajuri county, State of Minas Gerais, Brazil.

Sixteen semivariogram models were selected relating distance and pairwise sampling variation in damage by the different groups of insect species using low β_0_ values (closer to zero), higher β_1_ (closer to 1) and lower RSS (residual sum of squares), as the criteria for model selection. Of these initially selected models, eight were spherical, six exponential and two were Gaussian. The adjusted semivariogram models indicated spatial dependence of grain damage due to attack by the caterpillar, the cornsilk fly, and the grain beetles under field conditions (Table 3 and Fig 3). The degree of spatial dependence (DSD) of the damage and the presence of insect pests exhibited, for the most part, strong (GDE < 0.25) to moderate (0.25 < SDR < 0.75) spatial dependence (Table 3). The range of models adjusted for caterpillar damage varied from 9 to 10.50 m, for the cornsilk fly the ranged varied from 6.90 to 12.20 m, while for the square-necked beetle and the maize weevil the ranges varied from 51.90 to 170.70 m and from 10.70 to 80.40 m respectively (Table 3).

**Table 3:** Cross-validation by the kriging method of the values observed and estimated by the spherical, exponential and Gaussian models of grain injury by caterpillars (*Helicoverpa* spp. and *S*. *frugiperda*), cornsilk fly (*Euxesta* sp.), square-necked grain beetle (*C*. *quadricollis*), and maize weevil (*S*. *zeamais*) on Bt (Cry1Ab) and non-Bt corn.

Species/group	Model	β_1_	β_0_	RSS	C_0_	C_0_ + C	A_o_ (m)	R^2^	DSD	n
First season (Bt corn)										
Caterpillars	Spherical	0.26	151.35	5 x 10^7^	3000	50680	10.50	0.94	0.06	307
*Euxesta* spp.	Spherical	0.15	77.68	8326176	810	21260	10.00	0.28	0.04	307
*S*. *zeamais*	Gaussian	0.99	0.04	170	12.66	40.92	131.06	0.89	0.31	307
*C*. *quadricollis*	Exponential	0.67	1.45	297	9.33	57.24	15.90	0.84	0.16	307
First season (Non-Bt corn)										
Caterpillars	Spherical	0.16	177.46	1.59 x 10^8^	2200	48770	9.70	0.06	0.05	280
*Euxesta* spp.	Exponential	0.39	27.54	3926667	1040	8990	6.90	0.01	0.12	280
*S*. *zeamais*	Exponential	0.32	1.41	890	67.88	74.98	116.98	0.26	0.91	280
*C*. *quadricollis*	Spherical	0.64	1.87	705	2.34	48.99	14.32	0.15	0.05	280
Second season (Bt corn)										
Caterpillars	Exponential	0.12	197.07	2.0 x 10^7^	8400	58900	9.00	0.94	0.14	405
*Euxesta* spp.	Spherical	0.48	40.42	1.86 x 10^7^	1180	21620	10.70	0.02	0.05	405
*S*. *zeamais*	Gaussian	0.23	4.15	2626	98.99	169.94	170.67	0.76	0.58	405
*C*. *quadricollis*	Spherical	0.53	7.18	5600	7.10	295.76	10.70	0.02	0.02	405
Second season (Non-Bt corn)										
Caterpillars	Spherical	0.27	272.38	5.99 x 10^7^	5600	79510	10.10	0.04	0.07	368
*Euxesta* spp.	Spherical	0.36	22.52	2973253	260	7149	12.20	0.15	0.04	368
*S*. *zeamais*	Exponential	0.77	3.30	45449	250	788.10	51.90	0.71	0.32	368
*C*. *quadricollis*	Exponential	0.75	4.12	5902	187.50	375.10	80.40	0.76	0.50	368

In the header: β_0_ = intercept, β_1_ = slope, RSS = residual sum of squares, C_0_ = nugget effect, C = contribution, C_0_ + C = sill, A_o_ = range, DSD = degree of spatial dependence and n = number of point.

The maps of spatial distribution of grain damage by the caterpillars exhibited presented a prevalence of damage above 100, with a relatively uniform distribution in both corn genotypes (Fig 3). For the cornsilk fly, there was a prevalence of 20 to 100 injuries recorded and patches of aggregation with 100 to 500 injuries. Therefore, this species exhibits an uniform distribution, but with aggregation spots again in both, Bt and non-Bt corn (Fig 3). The injury density by the square-necked beetle in the first year of sampling ranged from 0 to 100 per sample, with predominantly densities ranging from 2 to 10 injuries and greater densities between corn rows than along the same corn row. In the second year of sampling, *C*. *quadricollis* density was from 10 to 20 injured grains per sample with aggregation patches of 20 to 100 injured grains. These results reflected the higher frequency of the beetle damage in the second year than in the first. In the second year, the density of this pest increased along the lines of cultivation (Table 3 and Fig 3). The maize weevil in Bt corn exhibited densities from 0 to 100 injured grains per sample, and aggregation at the left side of the crops with more movement towards the crop lines. In non-Bt corn, injury density ranged from 0 to 20 in the first and from 0 to 100 in the second year (Fig 3).

## Discussion

### Corn losses

High infestation levels of caterpillars, cornsilk flies, square-necked grain beetles, and maize weevils were observed on ears both in non-Bt and Bt corn crops sampled in this study. Damage caused by these pests was more than 77%, 22%, 63%, and 10% of corn ears evaluated, respectively. The total grain losses by arthropod pest species and also the losses by specific pest species differed between corn genotypes, and particularly so during the second season when the observed differences were greater.

The Bt corn used in this experiment constitutively expresses the Cry1Ab toxin that is effective in lepidopteran control, but not against flies (Diptera) and beetles (Coleoptera) [16,17]. The level of expression of this protein varies with the plant developmental phase and tissue with higher Cry1Ab toxin expression taking place during the vegetative growth and reducing afterwards, particularly during the reproductive stage. In addition, the Bt protein expression is higher in the leaves (9.35 μg/g of dry weight), and lower in the grains (0.31 μg/g of dry weight) and pollen (0.09 μg/g of dry weight) [36–38]. Thus, the high grain damage by caterpillars (> 57%) indicates confirms that the Bt toxin levels present in the grains are a rather weak defensive barrier against attacks by *S*. *frugiperda* and *Helicoverpa* spp. A similar result was found by Darvas et al. [9] and Burkness et al. [13], who observed low efficacy of the Cry1Ab toxin against *H*. *zea* and *O*. *nubilalis* on corn ears. However, as the Bt toxin Cry1Ab is not particularly effective against the fall armyworm (*S*. *frugiperda*) and resistance to this toxin have been reported for both armyworms and earworms in Brazil [39–41], both of these factors are also likely to be contributing for the small to modest differences in grain loss by caterpillar in Bt and non-Bt corn observed in our study.

The higher, although mild, damage of non-Bt corn by caterpillars observed in the second corn season may be due to the higher incidence of fall armyworm on the ears of non-Bt corn at this opportunity. This species is a notorious defoliator of corn plants in the Americas [42,43]. However, while the initial generations of *S*. *frugiperda* occurs in young corn plants, subsequent generations of this species infest older plants and at higher levels in subsequent cultivation cycles [44], damaging leaves, tassels, stems and ears, causing substantial loss of productivity [45,46].

The cornsilk fly presents a contrast with the caterpillars as it caused higher losses in Bt corn (first season = 22.1% and second season = 22.5%). These losses may have been influenced by the low susceptibility of this species to the Bt toxin and also because this species avoids ears attacked by earworms, as reported by Daly and Buntin [7] and confirmed in this research (Table 2). Females of cornsilk fly and earworms lay their eggs in the silk of corn ears with the first species avoiding oviposition on ears with eggs or larvae of the second [7,47,48]. Therefore, given that Bt corn was less attacked by caterpillars (Table 1 and Fig 1B), this likely favored egg-laying by the cornsilk fly.

Among the grain borer beetles, the square-necked grain beetle and the maize weevil were the most frequent. However, the losses caused by the former were higher than those caused by latter. Larvae and adults of the square-necked beetle are stored grain pests, but are also predators of other insect larvae, such as coffee berry borer [49]. As there were coffee plantations around the area where the experiment was carried out, this may have contributed to the greater damage caused by the square-necked beetle. In contrast, the losses by the maize weevil were the lowest among the most frequent pest species of our study. However, these losses by weevils can be considered high due to the destructive nature, high reproductive rate and the short generation time of *S*. *zeamais* [50] and the transport of this insect from the field into the storage units. While infestation begins in the field prior to harvest, the most serious damage (20–90% of grain weight loss) occurs during maize storage [51,52], especially in warmer climates and untreated maize [53]. The grain losses under these conditions are further intensified by the short generation time of the maize weevil [50]. The losses by this species here observed in the non-Bt corn were near twice higher than in the Bt corn and suspect that the attack caterpillars may favor the weevil incidence. The positive correlation between weevil and caterpillar attacks in our study lay credence for this perception.

### Spatial patterns

Studies of spatial distribution should faithfully reproduce the population parameters investigated. To this end, a minimum number of samples must be used to accurately determine the average value of the estimates [54]. In this study, the minimum number of samples required for suitable sampling was 280, and distributed at equidistant points. The sampling network had a direct influence on semivariogram adjustments, where low nugget effect (C_0_) and high sill (C_0_+C) values were observed. Regardless, the number of 280 samples allows the intended injury (and pest) estimation accounting for the associated spatial distribution.

The spatial distribution of pest injuries in Bt and non-Bt corn was similar, with a predominance of moderate to strong spatial dependence and aggregate distribution of injuries in the two corn genotypes. The aggregation pattern of distribution is common in insect populations [55], and this behavior provides a number of benefits over the life history of insects. Among these benefits are facility in finding mates, locating food and breeding sites, shelter, and relative protection against climatic conditions, action of insecticides and natural enemies [56,57]. The colonization pattern of Bt and non-Bt corn crops by armyworms and earworms were regular with a predominance of more than 100 damaged grains per sampling point. The homogeneity of ears damaged by these insects was reflected in the range of the semivariograms, with the difference between the largest and the smallest range being 1.5 m.

The production of sexl pheromone by the corn earworm (*Helicoverpa zea*) increases in the presence of corn plants and the males responded more actively to sexual pheromone in combination with volatiles of corn plants [58,59], increasing sexual encounters and consequently crop colonization. The majority of Lepidoptera species avoid laying their eggs on sites occupied by individuals of the same species or by other species with the same feeding habits. This behavior, besides of reducing the inter- and intra-specific competition, contributes to the distribution of these organisms in space [60]. Aggregation can also to be related to the larval dispersion of *H*. *zea* to adjacent plants [61].

The colonization of corn crops by cornsilk flies (*Euxesta* sp.) exhibit a regular pattern, with a predominance of areas with losses from 20 to 100 damaged grains per sampling point, but damage of 100 to 500 grains/sample exhibited an aggregated distribution. During the day, adults of the cornsilk fly are more abundant in the mid and basal areas of the plants, and at the end of the day the concentration is higher at the top of the plants [62]. Seal et al. [31] observed high aggregation of mated females of *Euxesta stigmatias* in the region near the ear of corn and oviposition peaks during the period from 9 am to 1pm. In addition, Kalsi et al. [62] observed high aggregations (Taylor's power law, Iwao's patchiness regression and Lloyd's patchiness) of *Euxesta* sp. near the corn ears during the developmental stages R2 and R3 (early reproductive stages). Cornsilk flies avoid laying eggs on plants attacked by corn earworms [7], a behavior may have also contributed to the higher incidence of this species and its damage with low incidence earworms in the fields.

The distribution pattern of both grain beetles, the square-necked (*C*. *quadricollis*) and the weevil (*S*. *zeamais*), was similar with predominance of high density patches bordered by areas with a gradual decrease of infestation. The occurrence of higher density patches (20 to 100 individuals) may be due to pheromone aggregation produced by these beetles [63]. In addition, synergism can occur between the volatiles of corn ears with sexual and aggregation pheromones enhancing insect responses to these compounds and favoring feeding, mate finding, and mating [64–66].

In summary, the pattern of insect losses in Bt corn is similar to that in non-Bt corn. Caterpillars are the main responsible these losses and their attack can intensify the losses caused by grain beetles, while also reducing the losses caused by the cornsilk fly. Moreover, our results provided geostatistical models with non-biased error estimations suitably adjusted for monitoring fall armyworms, earworms, cornsilk flies, and grain beetles. However, further studies are needed to spatially fine-tune management methods and strategies to reduce grain losses by insects in the field and grain storage.

## Supporting information

S1 Table Data for correlation analysis and comparisons of grain losses caused by insects in the Bt-corn and Non-Bt corn. SEASON: 1-first season and 2-second season; GENO: variety of maize grown in the research; BT: corn transgenic DKB 390 YG (expressing the Bt protein Cry1Ab), resistance to caterpillars; NON-BT: isoline DKB 390, susceptible to caterpillars; N: number of samples; TL: Total yield losses (%) caused by insects species exhibiting frequency of occurrence above 10% in the samples from both corn genotypes; CATH; yield losses (%) caused by *Cathartus quadricollis*; CATE: yield losses (%) caused by *Spodoptera frugiperda* + *Helicoverpa* sp.; EUXE: yield losses caused (%) by *Euxesta* spp.;MICE: yield losses (%) caused by mice; SITO: yield losses (%) caused by *Sitophillus zeamais*; CRYP: yield losses (%) caused by *Cryptolestes* sp.; TRIB: yield losses (%) caused by *Tribolium* sp.
(XLSX)
